# The Cecal Distribution of Microalgal Pigments in Rats: Do Carotenoids and Chlorophylls Play a Pharmacobiotic Role?

**DOI:** 10.3390/foods14132172

**Published:** 2025-06-21

**Authors:** Tatiele Casagrande do Nascimento, Patrícia Acosta Caetano, Marcylene Vieira da Silveira, Luiz Eduardo Lobo, Uashington Da Silva Riste, Mariany Costa Deprá, Maria Rosa Chitolina Schetinger, Cristiano Ragagnin de Menezes, Roger Wagner, Eduardo Jacob-Lopes, Leila Queiroz Zepka

**Affiliations:** 1 Food Science and Technology Department, Federal University of Santa Maria, Santa Santa Maria 97105-900, RS, Brazil; tatielecasagrande@gmail.com (T.C.d.N.); pati.caetano98@gmail.com (P.A.C.); uashington03@gmail.com (U.D.S.R.); rogerwag@gmail.com (R.W.);; 2 Department of Biochemistry and Molecular Biology, Federal University of Santa Maria, Santa Maria 97105-900, RS, Brazil

**Keywords:** bioactive pigments, gut microbiota, oleoresin supplementation, *Scenedesmus obliquus*, short-chain fatty acids

## Abstract

This study investigated the cecal distribution of lipophilic pigments (carotenoids and chlorophylls) from *Scenedesmus obliquus* and their effects on the activity of the intestinal microbiota in rats. Oleoresins containing different concentrations of microalgal pigments (from 0 to 600 µg·kg^−1^bw·d^−^^1^), previously characterized by chromatographic and spectrometric analyses, were administered for four weeks. At the end of the intervention, cecal content samples were collected and analyzed for their pigment composition, short-chain fatty acids (SCFAs), and probiotic microbiota. Nine pigments were identified in the cecal samples, with all-*trans*-zeaxanthin and pheophytin being the most abundant in all groups. Furthermore, 15-*cis*-lutein, all-*trans*-β-cryptoxanthin, and 9-*cis*-β-carotene—found exclusively in microalgal oleoresin—were detected only in animals receiving doses above 300 µg·kg^−1^bw.day^−1^, indicating a link with the SCFA modulation. These supplementations significantly increased the levels of acetate (300 and 450 µg·kg^−1^bw·d^−1^ −13% and 14%), butyrate (300 µg kg^−1^bw·d^−1^ −19%), and propionate (600 µg·kg^−1^bw·d^−1^ −16%). Notably, 300 µg·kg^−1^bw·d^−1^ significantly increased *Bifidobacterium* and *Lactobacillus* populations. Overall, the pigment supplementation positively influenced the gut microbiota composition and SCFA production in a dose-dependent manner, particularly at 300 µg·kg^−1^bw·d^−1^. These results support the potential application of microalgal pigments as functional food ingredients or supplements with gut health benefits.

## 1. Introduction

The gut microbiota is a central component of the intestinal ecosystem and fulfills critical functions in maintaining human well-being, including the protection against pathogenic microorganisms, modulation of the immune system, metabolic regulation, and absorption of nutrients and drugs. Imbalances in the gut microbiota are associated with a variety of gastrointestinal disorders. This microbial community can be affected by several variables, including the intake of compounds initially labeled as prebiotics, which are known to contribute to intestinal health maintenance [1,2].

However, the traditional definition of prebiotics—as indigestible food ingredients that support the host by specifically promoting the proliferation or function of specific bacteria—has recently been revised. The updated concept broadens the scope to include any fermentable compound capable of selectively promoting beneficial microorganisms within the gut microbiota, resulting in positive health outcomes. In this context, the term “pharmacobiotics” emerged to describe substances with therapeutic potential mediated by the modulation of the intestinal microbiota [3].

Non-digestible carbohydrates are the most extensively studied compounds in this field. However, bioactive phytochemicals such as carotenoids and chlorophylls—well known for their antioxidant properties—have been modestly explored regarding their potential role in promoting intestinal homeostasis [4,5,6].

Within this framework, microalgae such as *Scenedesmus obliquus*, rich in carotenoids and chlorophylls, stand out as promising functional sources of these compounds, particularly due to their demonstrated bioaccessibility, bioavailability, and bioactivity in oleoresin matrices, as shown in both in vitro and in vivo assays [7,8,9]. Nevertheless, studies evaluating the contribution of microalgae to gut health remain scarce and are largely limited to in vitro models or the use of the whole biomass [10,11].

Short-chain fatty acids (SCFAs), such as acetic, propionic, and butyric acids, are the main metabolic products of the intestinal microbiota—especially *Bifidobacterium* and *Lactobacillus*—and have been shown to support glucose and lipid metabolism, energy expenditure, and the regulation of immune responses and inflammation [12]. Consequently, SCFA levels serve as important markers of intestinal ecosystem health. However, no reports link the microalgal pigment intake to SCFA production. Specific activities on the levels of these cecal microbiota metabolites make the cecum a more optimized sampling site for determining environmental/dietary effects on the microbiota [13,14]

Given this background, the current research sought to examine the cecal distribution of microalgal carotenoids and chlorophylls in rats following a supplementation with oleoresin containing different concentrations of these pigments. Additionally, this study sought to evaluate their potential association with changes in the gut microbiota composition and SCFA production. This work contributes to addressing a knowledge gap regarding the uncertain ability of microalgal carotenoids and chlorophylls to exert direct “pharmacobiotic” effects.

## 2. Materials and Methods

### 2.1. Chemicals

All chemical compounds used for the BG-11 medium, sodium chloride, ammonium acetate (98%), tetrabutylammonium acetate, and all solvents for extraction and chromatography analysis were purchased from Sigma-Aldrich Chemical Co. (St. Louis, MO, USA). The standards all-*trans*-β-carotene, all-*trans*-lutein, and chlorophyll a (with purities ranging from 95.0% to 99.9%) were purchased from Sigma-Aldrich (Darmstadt, Germany).

### 2.2. Microalgal Strain and Biomass Production

The *S. obliquus* (CPCC05) strain was utilized for the production of whole-cell dried powder in a hybrid photobioreactor model, with the setup details, operating parameters, and downstream procedures previously outlined by Deprá et al. [15]. The biomass was isolated from the cultivation medium through centrifugation (Hitachi, Tokyo, Japan) at 1500× *g* for 10 min. The supernatant was discarded, and the remaining biomass was frozen at −18 °C for 24 h. Subsequently, it was freeze-dried for 24 h at −50 °C under a pressure below −175 μm Hg and then stored under refrigeration until further analysis.

### 2.3. Microalgal Pigments Extraction

The microalgal biomass (10 mg) was exhaustively extracted with 50 mL of dimethylformamide (DMF) saturated with magnesium carbonate (Mg_2_CO_3_). Afterward, the remaining biomass was re-extracted with 50 mL of methanol to exhaustion again. The obtained pigment extracts were mixed with 100 mL of diethyl ether and 400 mL of a 10% (*w*/*v*) sodium chloride solution (NaCl) in a separation funnel. The mixture was agitated and subsequently allowed to stand until the organic phase had fully separated. Then, the solvent phase was rinsed three times using 400 mL of a 10% NaCl solution (*w*/*v*). In sequence, pigments were transferred to diethyl ether and NaCl, and the above protocol was applied. The extracts were concentrated in a rotary evaporator under vacuum at 30 °C to dryness, and the dry residue was dissolved in the mobile phase for immediate HPLC analysis.

### 2.4. HPLC-PDA-MS/MS Pigments Analysis

The pigments were evaluated using high-performance liquid chromatography (HPLC) (Shimadzu, Kyoto, Japan) equipped with binary pumps (model LC-20AD), an inline degasser, and an automated sample injector (Rheodyne, Rohnert Park, CA, USA). The chromatographic system, featuring a photodiode array detector (PDA) (model SPD-M20A), was coupled in series to an atmospheric pressure chemical ionization (APCI) source (Shimadzu America, Columbia, MD, USA) and a Shimadzu 8040 triple quadrupole mass spectrometer. The pigments separation was performed on a C30 YMC column (5 μm, 250 × 4.6 mm) (Waters, Wilmington, DE, USA). HPLC-PDA analysis was performed in accordance with Rodrigues et al. [15]. Prior to HPLC-PDA analysis, the carotenoid extract was dissolved in a methanol (MeOH) and methyl tert-butyl ether (MTBE) solution at a 70:30 (*v*/*v*) ratio and filtered through Millipore membranes of 0.22 μm. The mobile phases—A (MeOH) and B (MTBE)—were obtained using a linear gradient program as follows: from 0 to 30 min 5% B; from 30 to 40 min, 5 to 30% B; from 40 to 41 min, 30 to 50% B; and from 41 to 50 min, 50 to 5% B. The flow rate was maintained at 0.9 mL·min^−1^, with an injection volume of 20 μL. The column temperature was controlled at 25 °C. UV-Vis spectra were acquired over the range of 220–700 nm, and chromatographic detection was carried out at 450 nm.

The MS/MS analysis was carried out in accord with Giuffrida et al. [16] with modifications; the APCI source operated in positive (+) ionization mode; detector voltage was set at 4.5 kV; interface temperature maintained at 350 °C; DL temperature at 250 °C; heat block temperature at 200 °C; nebulizing gas flow (N_2_) at 3.0 L·min^−1^; drying gas flow (N_2_) at 5.0 L·min^−1^; collision-induced dissociation (CID) gas pressure at 23 kPa (argon); and event time of 0.5 s. To enhance identification accuracy, MS/MS was simultaneously performed in SIM (Selected Ion Monitoring) and MRM (Multiple Reaction Monitoring) modes.

Identification was carried out based on the following combined criteria: elution order on a C30 HPLC column, co-chromatography with authentic standards, UV-Vis spectrum, and mass characteristics (protonated molecule ([M+H]+) and MS/MS fragments). And it was compared with information reported in the literature [9,17,18,19,20]. Individual quantification of the pigment content was performed by HPLC-PDA using calibration curves constructed from five-point concentration. Analytical curves of all-*trans*-lutein, all-*trans*-β-carotene, and chlorophyll *a* were employed to quantify the xanthophylls, carotenes, and chlorophylls, respectively. Results were expressed as micrograms per gram of sample (µg·g^−1^).

### 2.5. In Vivo Experimental Design

This study was approved by the Animal Ethics Committee (CEUA) of the Federal University of Santa Maria under number 2662070222. Male Wistar rats (150–250 g) were obtained from the Animal Facility of the Federal University of Santa Maria and kept in the Pharmacology sector animal facility. The animals were kept under controlled temperature (23 ± 1 °C), light–dark cycle (12/12 h), and humidity conditions (50 ± 10%), with free access to water and commercial diet Puro Lab 22PB^®^ (see composition in Table 1). The animals were randomly distributed into six groups (n = 10 per group). The sample n value was determined according to the test curve of Dressler et al. [21]. The control group received 300 µL of 0.9% saline solution by oral gavage (without pigments), while the other groups received different doses of microalgal pigments suspended in sunflower oil (oleoresin), at concentrations of 75, 150, 300, 450, and 600 µg kg^−1^ day^−1^, respectively. Given the lack of official guidelines on the recommended daily intake of microalgal pigments, the doses used in this study were selected based on concentrations previously shown to be safe and beneficial in eutrophic animal models using lipophilic *S. obliquus* extracts [7]. These doses were then converted for use in rats through allometric scaling, using a JavaScript-based calculator (http://clymer.altervista.org/minor/allometry.html, accessed on 15 April 2025) that estimates interspecies dosages by accounting for body weight differences on an exponential scale. After four weeks, the animals were euthanized with 100% isoflurane (DCB no.: 05082/CAS no.: 26675-46-7). The intestine was retired, and fecal samples (cecum) were collected to analyze pigments, short-chain fatty acids, and total probiotic microbiota.

#### 2.5.1. Eutrophic Status

To assess the eutrophy of the animals, water intake (mL) and feed intake (g) were measured daily. Body weight (g) was recorded on days 1, 3, 7, 10, 14, 17, 21, and 28 of the experiment. After euthanasia, abdominal fat was collected and weighed (g). Additionally, the animals were monitored throughout the experiment for any clinical signs of toxicity.

#### 2.5.2. Determination of Pigments in Commercial Diet and Fecal Samples

The pigments in the commercial diet (1 g) and fecal samples (1 g) were extracted and analyzed according to Section 2.3 and Section 2.4, respectively. Quantitative results were shown as micrograms per gram of sample (µg·g^−1^).

#### 2.5.3. Determination of Short-Chain Fatty Acids (SCFAs) and Total Microbiota (*Bifidobacterium* and *Lactobacillus*)

The microbiota samples were thawed to 5 °C and agitated manually to homogenize them. Then, 250 mg of samples and 750 µL of methanol were added into a 1.5 mL polypropylene microtube and then shaken with a vortex for 1 min. Then 250 μL of the supernatant was removed and transferred to a new microtube containing 250 μL of formic acid. The mixture was manually stirred and subjected to centrifugation for 3 min. After centrifugation, 250 μL of the supernatant of the mixture was collected in another polypropylene tube previously containing 500 μL of isoamyl alcohol solution (692.40 μg mL^−1^ in methanol), used as an internal standard, and was homogenized and centrifuged again. Then, 650 μL of sample was inserted into a 2 mL injection vial, and 1 μL of extract was injected into a gas chromatograph equipped with a flame ionization detector (GC-FID; Varian Star 3400, Palo Alto, CA, USA) and an autosampler (Varian 8200CX, Palo Alto, CA, USA) in split mode (1:10) at 250 °C. Hydrogen was employed as the carrier gas at a constant pressure of 12 psi. The analytes (acetic, propionic, butyric, valeric, and isovaleric acids) were resolved using a CP-Wax 52CB capillary column (50 m × 0.32 mm; 0.20 μm film thickness of the stationary phase). The initial column temperature was set at 60 °C for 1 min and increased to 90 °C at a rate of 10 °C min^−1^, then to 110 °C for 5 °C min^−1^, and, lastly, to 230 °C at a rate of 20 °C min^−1^, where it remained for 1 min. The detector was operated at a temperature of 250 °C. Method validation included the evaluation of selectivity, linearity, linear range, repeatability, precision, limit of detection (LOD), and limit of quantification (LOQ) for acetic, propionic, butyric, and isovaleric acids. Linearity was determined by constructing a regression equation based on the least squares method. LOD and LOQ values were obtained by sequential dilutions up to signal-to-noise ratios of 3:1 and 6:1, respectively. Precision was assessed by analyzing the repeatability of six replicated samples. Accuracy was evaluated by measuring the recovery of known quantities of standard compounds spiked into a diluted sample. The results were expressed in mol 100 mol^−1^ of each SCFA in the samples.

For determination of microbiota, serial dilutions were transferred to triplicate sterile Petri plates containing MRS agar (Himedia Curitiba, Paraná, Brazil), for total *Bifidobacterium* and *Lactobacillus* counts. Plates were incubated at 37 °C for 72 h. The dilution of the microparticles involved weighing 1 g of microparticles and adding 9 mL of sterile phosphate buffer solution (pH 7.5), according to the procedure outlined by Sheu et al. [22]. Results were shown as log colony-forming units per gram (log CFU·g^−1^).

### 2.6. Statistical Analysis

Statistical analysis was performed using GraphPad Prism 5.0 software (GraphPad Software Inc., La Jolla, CA, USA). Data normality criteria were assessed by the Shapiro–Wilk test. Subsequently, the significance of the experimental data was determined using one-way ANOVA followed by Tukey’s test (*p* < 0.05). Data were expressed as mean ± standard deviation of the mean (SMD).

## 3. Results and Discussion

### 3.1. Eutrophic Status Analysis

**Figure 1:**
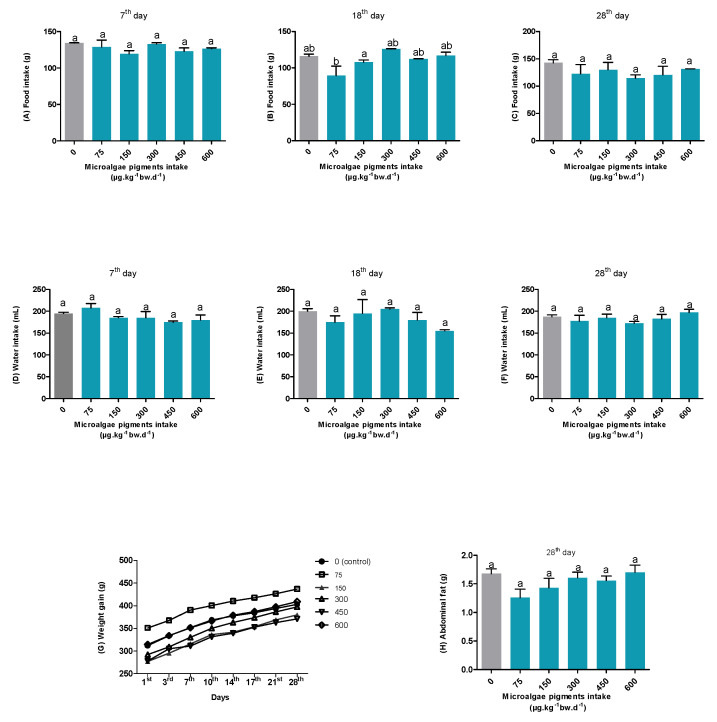
The food intake (**A**–**C**), water intake (**D**–**F**), body weight (**G**), and abdominal fat (**H**) of rats. Different letters between the columns indicate statistical differences (*p* < 0.05).

Data on the food and water intake, body weight, and abdominal fat were collected to analyze the rats’ eutrophy status in the experimental period. As shown in Figure 1, the food intake (A, B, and C) among the groups that received different concentrations of microalgal pigments (0, 75, 150, 300, 450, and 600 µg·kg^−1^bw·d^−1^) did not differ throughout the experiment, except for the 75 µg·kg^−1^·bw·d^−1^ group on the 18th day. However, by day 28, this difference was no longer observed. Likewise, no statistical differences were found between the groups for water intake (Figure 1D–F). Furthermore, Figure 1G shows that the body weight gain in the experimental groups followed the same variation pattern as the control group (0 µg·kg^−1^bw·d^−1^) throughout the experiment (from day 1 to day 28).

The abdominal fat content was also evaluated (Figure 1H), given the purely lipophilic nature of the extract administered—which was rich in carotenoids—which could influence the lipid accumulation metabolism [23]. Nevertheless, no statistically significant differences were observed among the groups at the end of the experiment.

The compilation of these results indicates that the different concentrations of carotenoids and chlorophylls from *S. obliquus* provided in the oleoresin during the study did not interfere with the healthy development or growth of the rats. Moreover, no clinical signs of toxicity or mortality were observed throughout the entire experiment.

### 3.2. Carotenoids and Chlorophylls Identification by HPLC-PDA-MS/MS

The chromatographic and spectrometric characteristics of carotenoids and chlorophylls analyzed in the present study (oleoresin containing the lipophilic extract of *S. obliquus*, commercial diet, and fecal content samples) are presented in Table 2. The pigment identification was based on the chemical evidence obtained through the chromatographic analysis, including the elution order, UV-visible features, and MS/MS experiments, which confirmed the assignment of the protonated molecule ([M+H]^+^) to the peaks identified through the expected fragments for each compound. Furthermore, the identification was based on a detailed description previously reported in the literature [9,17,18,20,24].

Twenty-two pigments were identified throughout the trial (sixteen carotenoids and six chlorophylls). Of these compounds, twenty-one were present in the microalgal oleoresin, including sixteen carotenoids (all-*trans*-violaxanthin—peak 1, 9-*cis*-violaxanthin—peak 2, 15-*cis*-lutein—peak 3, 13-*cis*-lutein—peak 6, all-*trans*-lutein—peak 7, all-*trans*-zeaxanthin—peak 8, 9-*cis*-lutein—peak 10, canthaxanthin—peak 11, 9-*cis*-zeaxanthin—peak 13, 2′-dehydrodeoxymyxol—peak 14, 5,6-epoxy-β-carotene—peak 15, all-*trans*-β-cryptoxanthin—peak 16, all-*trans*-echinenone—peak 17, 9-*cis*-echinenone—peak 19, all-*trans*-β-carotene—peak 20, and 9-*cis*-β-carotene—peak 22) and five chlorophylls (hydroxychlorophyll—peak 4, chlorophyll *b*—peak 5, chlorophyll *a*—peak 9, chlorophyll *a*′—peak 12, and pheophytin *a*—peak 21). This pigment profile is a recurring characteristic of the taxonomic group of green algae, such as *S. obliquus* [25,26]. Moreover, the profile found is similar to previous characterizations carried out for this same microalga [9,17,19].

In contrast, only five compounds (three carotenoids and two chlorophylls) were detected in the commercial diet, all-*trans*-lutein—peak 7, all-*trans*-zeaxanthin—peak 8, 9-*cis*-zeaxanthin—peak 13, hydroxypheophytin *a*—peak18, and pheophytin *a*—peak 21, and were probably xanthophylls and chlorophylls from ingredients that make up the commercial diet, such as corn and soy, for example [27,28]. Likewise, these pigments were also detected in fecal samples from the control group and the groups that ingested 75 and 150 µg·kg^−1^bw·d^−1^ of pigments.

In addition to these five compounds, the 300 and 450 groups also showed the 15-*cis*-lutein (peak 3), 9-*cis*-lutein (peak 10), all-*trans*-β-cryptoxanthin (peak 16), and 9-*cis*-β-carotene (peak 22), totaling nine pigments. Similarly, these nine pigments were detected in the fecal content of animals that received a daily dose of 600 µg·kg^−1^bw·d^−1^. Still, for this same dosage, the isomer 13-cis-lutein (peak 6) was detected, increasing the total to ten pigments.

It is worth noting that only in the fecal content of the groups that received daily concentrations above 300 µg·kg^−1^bw·d^−1^ of pigments was it possible to detect isomers of lutein, 9-*cis*-β-carotene, and all-*trans*-β-cryptoxanthin. Among these, the latter two are present only in the *S. obliquus* oleoresin, which indicates that this is the source of their accumulation. In contrast, hydroxypheophytin *a*, which is absent in the microalgal oleoresin, was detected in all experimental groups. Although this chlorophyll derivative is present in the commercial diet, its presence in the fecal content may result from microbial biotransformations occurring in the intestine [5]. Similarly, it has been proposed that *cis* isomers of carotenoids may also originate from gastrointestinal conditions [29].

### 3.3. Carotenoids and Chlorophylls Quantification

The carotenoid and chlorophyll content (µg·g^−1^) of the *S. obliquus* oleoresin, commercial diet, and fecal content samples from the experimental trial (groups 0 (control), 75, 150, 300, 450, and 600 µg·kg^−1^bw·d^−1^) is presented in Table 3.

Considering the carotenoid profile, all-*trans*-lutein (622.98 ± 1.20 µg·g^−1^) followed by all-*trans*-zeaxanthin (290.13 ± 0.33 µg·g^−1^) and all-*trans*-β-carotene (264.66 ± 0.27 µg·g^−1^) were the major constituents in the microalgal oleoresin. With the exception of all-*trans*-β-carotene, the same occurred in the commercial diet (all-*trans*-lutein: 1.60 ± 0.25 µg·g^−1^ and all-*trans*-zeaxanthin: 1.46 ± 1.10 µg·g^−1^). Despite this, all-*trans*-zeaxanthin was the most abundant carotenoid in the cecal content of the experimental groups, totaling 1.52 ± 1.03, 1.65 ± 1.60, 2.03 ± 0.23, 1.80 ± 1.78, 2.48 ± 1.09, and 3.15 ± 1.10 µg·g^−1^ for the control, 75, 150, 300, 450, and 600 groups, respectively.

Pigments such as 15-*cis*-lutein (0.20 ± 0.24, 0.21 ± 0.31, and 0.22 ± 0.99 µg·g^−1^), all-*trans*-β-cryptoxanthin (0.27 ± 0.51, 0.28 ± 0.69, and 0.42 ± 0.50 µg·g^−1^), and 9-*cis*-β-carotene (0.19 ± 0.92, 0.21 ± 0.31, and 0.32 ± 0.60 µg·g^−1^) were quantifiable only in animals supplemented with higher pigment concentrations (300, 450, and 600 µg·kg^−1^bw·d^−1^). In addition, 13-*cis*-lutein was detected exclusively in the 600 µg·kg^−1^ group (0.21 ± 0.30 µg·g^−1^). This fact reinforces the idea that the presence of several carotenoids in higher concentrations tends to decrease the intestinal cellular uptake of some structures, and consequently these compounds follow the intestinal transit [30,31].

Considering a previous study of in vitro intestinal digestion and absorption, 15-*cis*-lutein, all-*trans*-β-cryptoxanthin, and 9-*cis*-β-carotene showed a good bioaccessibility (74%, 36%, and 22%) but were not satisfactorily absorbed by Caco-2 cells in the intestinal model [18], which may justify their presence in the fecal samples analyzed in this study. On the other hand, canthaxanthin, despite presenting a 99% bioaccessibility and also not having been absorbed by the intestinal mucosa in the same in vitro study, was not detected in the fecal content of the present study, which is consistent with the possible degradation, bioconversion, or microbial metabolism during intestinal transit. The same may have occurred for the other carotenoids present in the oleoresin and not detected in the analyzed samples.

**Figure 2:**
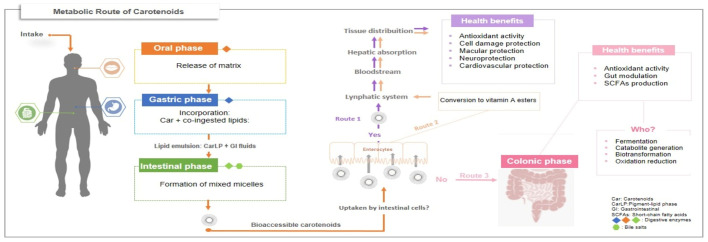
Metabolic routes of carotenoids after intake.

As illustrated in Figure 2, carotenoids, once ingested, are released from the food matrix through mechanical and enzymatic actions during digestion. Due to their lipophilic nature, they associate with co-ingested lipids, forming emulsions that are subsequently digested by lipases with the aid of bile salts in the small intestine. Lipid digestion leads to the formation of mixed micelles—structures responsible for solubilizing and transporting carotenoids through the aqueous environment of the intestine to the enterocytes. In this form, carotenoids become bioaccessible, meaning they are available for absorption by intestinal cells [32].

From this point, three potential routes may be followed: Route 1—carotenoids are absorbed and transported via chylomicrons into the lymphatic system, then to the liver, where they may be stored, metabolized, or redistributed to target tissues to exert health-promoting effects. Route 2—provitamin A carotenoids are converted into retinyl esters within enterocytes before entering the bloodstream. Route 3—unabsorbed carotenoids proceed to the colon, where they may be beneficially utilized by the gut microbiota [6,33]. Once in the colon, possible microbial processing pathways include catabolism, fermentation, or biotransformation.

To date, the specific mechanisms governing carotenoid–microbiota interactions remain unclear, and few studies have directly or indirectly investigated this relationship. An investigation conducted by Kaulmann et al. [34] demonstrated the generation of novel compounds during fermentation by the intestinal microbiota and inferred that tetraterpenoids were effectively metabolized. It is known that carotenoids can be cleaved by oxygenases present in various organisms, including those in the gut microbiome, supporting the hypothesis that unabsorbed compounds may be locally metabolized by gut microbes [35]. Among the resulting metabolites, apocarotenoids have been identified as potential modulators of the gut microbiota, contributing to intestinal health benefits [36].

Regarding the chlorophyll fraction, although chlorophyll *a* was the main tetrapyrrole in the oleoresin (4459.81 ± 2.17 µg·g^−1^), at the end of the in vivo administrations, pheophytin *a* was more abundant in the cecal content of all groups. Furthermore, an increase was observed in the following order 1.75 ± 0.86, 1.86 ± 0.38, 2.03 ± 1.98, 2.51 ± 0.39, and 3.17 ± 0.19 µg·g^−1^ for groups 75, 150, 300, 450, and 600, respectively.

**Figure 3:**
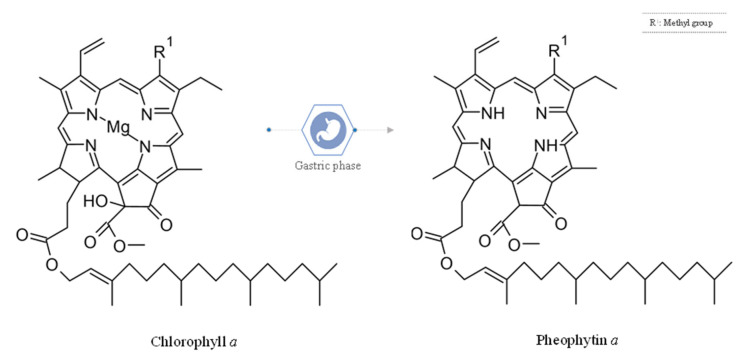
Chemical structure of chlorophyll *a* and pheophytin *a*.

It is believed that the pheophytin content may originate from the loss of the central magnesium from the chlorophyll a structure (see Figure 3). This transformation is common due to the structural vulnerability of chlorophyll at an acidic pH, which is a normal biological condition of the gastric phase (pH close to 3.0) during the digestive process [9].

Other hypotheses arise when observing the content of chlorophyll derivatives present in the control group. Although this group exclusively consumed the commercial diet, which contained reduced amounts of hydroxypheophytin *a* (0.18 ± 0.51 µg·g^−1^) and pheophytin *a* (0.14 ± 0.82 µg·g^−1^), it is notable that the fecal samples presented significantly higher concentrations of these compounds—approximately twelve times more pheophytin a and twice as much hydroxypheophytin *a*. Thus, the accumulation of these derivatives in the cecum would be another tangible hypothesis. However, the free access to the commercial diet during the experimental trial weakens this possibility. According to Li et al. [5], there is also the possibility of chlorophylls being biotransformed during the in vitro digestion or colonic fermentation.

Analyzing the totality, the twenty-one compounds identified in the oleoresin of *S. obliquus* amounted to 7329.09 ± 1.56 µg·g^−1^ of pigments. Of this amount, approximately 68% (4979.45 ± 2.28 µg·g^−1^) and 32% (2349.77 ± 1.11 µg·g^−1^) corresponded to chlorophylls and carotenoids, respectively. On the other hand, the compounds found in the commercial diet totaled 4.17 ± 0.68 µg·g^−1^, which is approximately two thousand times lower than the content contained in the microalgal oleoresin. Furthermore, unlike microalgal oleoresin, the largest fraction of pigments found in the diet was carotenoids (3.85 ± 0.57 µg·g^−1^), while chlorophylls accounted for approximately 8% (0.32 ± 0.55 µg·g^−1^).

In contrast, in fecal samples the proportion varied according to the concentration ingested. Interestingly, the group that did not ingest pigments (control) presented approximately 50% carotenoids and chlorophylls. In the experimental groups (75, 150, 300, 450, and 600), the total amount of carotenoids (55–66%) stood out in relation to chlorophylls (35–45%). This increase in the relative proportion of carotenoids compared to chlorophylls can be attributed to the higher carotenoid content in the oleoresin, combined with the lower structural stability of chlorophylls, which are more prone to degradation due to the pH variation, oxidative environment, and enzymatic reactions [37].

In general, a dose-dependent relationship was observed in the global distribution of carotenoids and chlorophylls in the fecal content, since the concentration of these pigments in the cecum increased proportionally to the amount present in the administered oleoresin.

### 3.4. SCFA Levels and Microbiota

**Figure 4:**
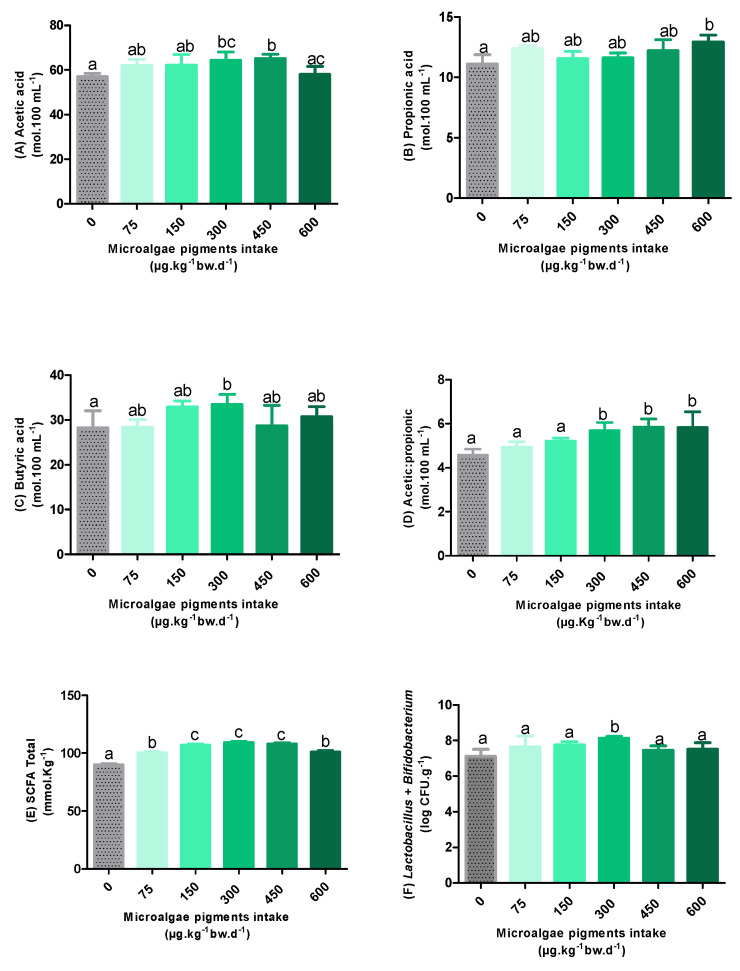
The short-chain fatty acid content (**A**–**E**) and total microbiota (**F**) after the intake of oleoresin with different concentrations of microalgae pigments. Different letters between the columns indicate statistical differences (*p* < 0.05).

The results regarding the SCFA profile, as well as the total microbiota, obtained from the cecal samples of the experimental groups are presented in Figure 4.

As shown in Figure 4A, acetic acid levels in the groups supplemented with 300 and 450 µg·kg^−1^ bw·d^−1^ increased by 13% and 14%, respectively, compared to the control group, showing statistically significant differences (*p* < 0.05). The other groups did not differ statistically from the control. However, a significant reduction of 11% in acetic acid levels was observed for the supplementation of 600 µg·kg^−1^ bw·d^−1^, compared to that of 450 µg·kg^−1^ bw·d^−1^ of pigments.

Among all the AGCCs analyzed in this study, acetic acid was the most abundant. This finding is relevant, since high levels of acetate are associated with beneficial effects, including a decreased appetite, improved glucose metabolism, reduced inflammatory processes, and lipid accumulation in adipose and liver cells [3,38].

Regarding propionate levels (Figure 4B), only the animals that received 600 µg·kg^−1^ bw·d^−1^ of microalgal pigments showed a statistically significant difference compared to the control group (*p* < 0.05), with an increase of 16%. The other experimental groups did not differ significantly from each other regarding this SCFA.

In contrast, in relation to butyric acid levels (Figure 4C), a statistically significant increase (19%) was observed only in animals supplemented with 300 µg·kg^−1^bw·d^−1^ of microalgal pigments (*p* < 0.05). As observed for propionic acid levels, there were no statistical differences between the other experimental groups.

When analyzing the acetic acid/propionic acid ratio (Figure 4D), it is observed that no concentration of pigments provided to the animals presented a statistically significant difference from the control (*p* < 0.05). Considering that the acetic acid/propionic acid ratio is an important indicator of the functional balance of the intestinal microbiota [39], the similarity observed in relation to the control group proposes the maintenance of this balance.

Overall, an overall increase of 11–21% in SCFA levels (Figure 4E) was observed in the pigment-supplemented groups, compared to the control group. This increase indicates a positive modulation of the intestinal microbial activity. According to Agus et al. [40], the increase in the levels of these metabolites is essential because, in addition to regulating the immune system and inflammatory responses, they can also contribute to improving the glycemic and lipid metabolism, as well as controlling the energy intake.

Despite the significant increase compared to the control, a downward trend in total levels was observed after the supplementation with 300 µg/kg body weight·d^−1^, being more pronounced at the concentration of 600 µg·kg^−1^bw·d^−1^, which presented a statistically significant difference among the groups of 150, 300, and 450 µg·kg^−1^bw·d^−1^. This corroborates the evidence that higher concentrations of pigments may be associated with a reduction in the abundance of the intestinal microbiota. This assumption is plausible since high doses of carotenoids can exert a pro-oxidant effect, which results in cellular damage that compromises the microbial activity. However, the lack of a broader microbiome profile limits the robustness of this hypothesis. A more accurate interpretation could be achieved in future studies through the inclusion of genetic sequencing, intestinal histology, inflammatory cytokine measurements, and gut permeability markers.

The data related to the total microbiota (Figure 4F) showed a significant increase in the microbial population only in the group treated with the oleoresin containing 300 µg·kg^−1^bw·d^−1^ of pigments, which reinforces the potential prebiotic effect of this concentration. However, it is important to highlight that the microbiota analyzed in this study refers exclusively to the total *Bifidobacterium* and *Lactobacillus*. This indicates that the significant increases in SCFA levels observed in groups 450 (acetic acid) and 600 (propionic acid) may be associated with the activity of other bacterial groups not contemplated in the quantification performed, such as the phylum Bacteroidetes (acetic acid), *Clostridium ramosum* (propionic acid), and *Bacteroides thetaiotaomicron* (acetic acid/propionic acid) [41].

In support of the results of this study, Table 4 presents comparisons between pigments of a non-microalgal origin and biomass. Previous research demonstrates that pigments such as β-carotene and lutein from other sources contributed to intestinal homeostasis. Scientific evidence indicates that xanthophylls like lutein and zeaxanthin exert a more pronounced effect on gut microbiota modulation compared to carotenes [42]. Nevertheless, β-carotene has also shown significant contributions to intestinal homeostasis by increasing the abundance of *Bacteroidetes* and *Proteobacteria*, while reducing harmful bacteria and promoting the growth of beneficial strains such as *Bifidobacterium*, *Collinsella* (at intermediate doses), and *Lactobacillus* (at higher doses) [43]. Lycopene, another carotene, has also demonstrated the ability to inhibit the Proteobacteria proliferation and increase the abundance of *Bifidobacterium and Lactobacillus* [44].

In addition, diets rich in fruits and vegetables with high carotenoid contents have been associated with increased levels of SCFAs in the gut and feces [45,46]. Consistent with the results of the present study, findings from Rocha et al. [6] reported a marked increase in butyrate production following a carotenoid administration. Other studies have also demonstrated that carotenoids contribute to intestinal immune homeostasis through the regulation of the immunoglobulin A (IgA) production and the prevention or delay of dysbiosis [48].

Studies on the influence of β-cryptoxanthin on the gut microbiota remain limited. The only available investigation found no significant changes after eight weeks of supplementation with 3 mg·d^−1^ [47]. Similarly, the evidence regarding chlorophylls is scarce. However, the negative regulation of the Firmicutes phylum and the positive modulation of Bacteroidetes was observed [4]. Additionally, the supplementation with a chlorophyll-rich extract was found to increase the Akkermansia abundance, a microorganism associated with the glucose metabolism [49].

Among the few studies available on microalgae, the reported beneficial effects have mainly been attributed to whole biomass consumption. Spirulina, for example, was recently investigated for its potential to stimulate the production of short-chain fatty acids (SCFAs), such as acetate, propionate, butyrate, hexanoate, isovalerate, and isobutyrate, and to promote the growth of beneficial bacteria, like *Lachnospiraceae*, *Lactobacillus*, and *Bifidobacterium*. However, these effects have been largely attributed to the fiber and phenolic compounds naturally present in the biomass [50].

Unlike prebiotic carbohydrates, whose benefits are primarily derived from microbial fermentation, the beneficial effects observed with pigments are also attributed to their accumulation in the intestinal lumen. This local presence may enhance antioxidant activity and positively influence the composition and functionality of the gut microbiota [52,53]. Additionally, pigments may indirectly affect the gut microbiota by modulating the gastrointestinal transit time, the pH, and the synthesis and release of antimicrobial peptides and secretory immunoglobulins, all of which play a crucial role in maintaining intestinal homeostasis [36].

Overall, the microalgal pigments present in the *S. obliquus* oleoresin were detected in the cecal content and demonstrated the ability to stimulate SCFA production and promote the growth of *Bifidobacterium* and *Lactobacillus*, indicating a positive effect on the gut microbiome, especially at concentrations of 300, 450, and 600 µg·kg^−1^bw·d^−1^. Furthermore, the data suggest that this beneficial effect may be associated with the presence of specific carotenoids, such as all-*trans*-β-cryptoxanthin, 15-*cis*-lutein, and 9-*cis*-β-carotene, since these compounds were quantified exclusively in the groups supplemented with oleoresin. To the best of our knowledge, this is the first study to evaluate such effects using pigments extracted from microalgae, thereby expanding our understanding of their functional potential. Combined with the already well-established antioxidant properties of these compounds, this set of effects highlights the multifunctional role of S. obliquus in health promotion. This functional versatility underscores the value of this microalga as a promising bioactive matrix whose integrated physiological impact warrants further investigation.

## 4. Conclusions

The results of this study provide relevant insights into the interactions between microalgal pigments and the activity of the intestinal microbiota. The supplementation with oleoresins containing different concentrations of *S. obliquus* pigments did not adversely affect the eutrophy of the animals. All-*trans*-zeaxanthin and pheophytin *a* were the most abundant pigments in all experimental groups. Additionally, pigments exclusive to microalgal oleoresin, such as 15-*cis*-lutein, all-*trans*-β-cryptoxanthin, and 9-*cis*-β-carotene, were detected only in animals that received doses equal to or greater than 300 µg·kg^−1^bw·d^−1^, which proposes a possible association with the modulation of SCFA levels. These supplements promoted significant increases in the levels of acetate (300 and 450 µg·kg^−1^bw·d^−1^), butyrate (300 µg·kg^−1^bw·d^−1^), and propionate (600 µg·kg^−1^bw·d^−1^). In addition, the dose of 300 µg·kg^−1^bw·d^−1^ significantly increased the populations of *Bifidobacterium* and *Lactobacillus*. In general, the supplementation positively influenced both the composition of the intestinal microbiota and the production of SCFAs, in a dose-dependent manner, with an emphasis on the dose of 300 µg·kg^−1^bw·d^−1^. This supports the possibility that pigments may function as pharmacobiotic modulators, with positive implications for intestinal health. The differential distribution of carotenoids in the cecal contents and the conversion or accumulation of chlorophylls into derivatives, such as pheophytins, indicate that gastrointestinal transit and the microbiota play a central role in the transformation or elimination of these compounds, which deserves further investigation. Taken together, these findings support the use of microalgal pigments as promising functional ingredients in foods or supplements aimed at promoting intestinal health.

## Figures and Tables

**Table 1:** The composition of the commercial diet Puro Lab 22PB^®^.

Composition *
Folic acid
Nicotinic acid
Antioxidant additive (BHA and BHT)
Biotin
Calcitic limestone
Choline chloride
Sodium chloride (Common salt)
Soybean meal (GMO from Agrobacterium SP.)
Wheat bran
Dicalcium phosphate
Calcium iodate
Lysine
Methionine
Ground whole corn (GMO from *Bacillus thuringiensis*, *Streptomyces* sp.)
Calcium pantothenate
Sodium selenite
Ground whole soybean (GMO from *Agrobacterium* sp./Pressure treatment)
Cobalt sulfate
Copper sulfate
Iron sulfate
Manganese sulfate
Zinc sulfate
Whole wheat
Vitamin A, B1, B12, B2, B6, C, E, K3

* Source: https://www.purotrato.com.br/produtos/Puro-Lab-22PB accessed on 15 April 2025.

**Table 2:** Chromatographic characteristics, UV-vis spectrum, and mass obtained by HPLC–PDA–MS/MS of pigments from *S. obliquus* oleoresin, commercial diet, and cecal samples from rats after ingestion of oleoresin with different concentrations of microalgae pigments.

Peak	Pigments	tR (min) ^a^	UV–Vis Characteristics			Fragment Ions (Positive Mode) (*m*/*z*)	
			λmáx (nm) ^b^	III/II (%) ^c^	AB/II (%) ^d^	[M+H]^+^	MS/MS
1	all-*trans*-violaxanthin	8.1	415,438,468	85	0	601	583 [M+H–18]^+^, 565 [M+H–18–18]^+^, 509 [M+H–92]^+^
2	9-cis-violaxanthin	9.0	326, 411, 435,464	72	18	601	584 [M+H–18]^+^, 565 [M+H–18–18]^+^, 509 [M+H–92]^+^
3	15-*cis*-lutein	11.2	328,412,439,464	16	24	569	551 [M+H–18]^+^, 533 [M+H–18–18]^+^
4	hydroxychlorophyll *a*	11.2	430, 664	na ^e^	na	909	631 [M+H–278]^+^
5	chlorophyll *b*	11.7	465,654	na	na	907	629 [M+H–278]^+^, 569 [M+H–278–60]^+^
6	13-*cis*-lutein	12.4	415,440,465	37	nc ^f^	569	551 [M+H–18]^+^, 533 [M+ –18–18]^+^
7	all-*trans*-lutein	13.6	420, 445, 472	50	0	569	551 [M+H–18]^+^, 533 [M+H–18–18]^+^
8	all-*trans*-zeaxanthin	15.8	425,451, 476	22	0	569	551 [M+H−18]^+^, 533 [M+H−18−18]^+^, 495, 477 [M+H−92]^+^, 459
9	chlorophyll *a*	15.7	431, 664	na	na	893	615 [M+H−278]^+^, 583 [M+H−278−31]^+^, 555 [M+H−278–59]^+^
10	9-*cis*-lutein	16.4	418, 440, 470	50	nd ^g^	569	550 [M+H−18]^+^, 533 [M+H−18−18]^+^
11	canthaxanthin	17.0	460	nc	nd	565	547 [M+H−18]^+^
12	chlorophyll *a′*	17.4	430, 664	na	na	893	615 [M+H−278]^+^, 583 [M+H−278−31]^+^, 555 [M+H−278–59]^+^
13	9-*cis*-zeaxanthin	20.9	420, 446, 471	25	nc	569	551 [M+H−18]^+^, 533 [M+H−18−18]^+^, 495, 477 [M+H−92]^+^, 459
14	2′-dehydrodeoxymyxol	22.0	445,474,504	63	0	567	549 [M+H−18]^+^
15	5,6-epoxy-β-carotene	22.9	420,446,471	50	0	553	535 [M+H−18]^+^, 461 [M+H−92]^+^, 205
16	all-*trans*-β-cryptoxanthin	24–24.6	425,450,476	25	0	553	535 [M+H−18]^+^
17	all-*trans*-echinenone	25.1	461	nc	0	551	533 [M+H−18]^+^, 427, 203
18	hydroxypheophytin *a*	26–27.2	408,666	na	na	887	869 [M+H−18]^+^; 803 [M+H−63]^+^; 609 [M+H−278]^+^; 591 [M+H−278−18]^+^; 531 [M+H−278-18−60]^+^
19	9-*cis*-echinenone	27.6	454	nc	nc	551	533 [M+H−18]^+^, 427, 203
20	all-*trans*-β-carotene	33.6	425, 451, 476	25	0	537	444 [M+H−92]^+^, 399, 355
21	pheophytin *a*	34.4–36.0	408,666	na	na	871	593 [M+H−278]^+^; 533 [M+H−278−60]^+^
22	9-*cis*-β-carotene	36.6	425, 451, 476	30	nc	537	444 [M+H−92]^+^, 399, 355

^a^: The retention time (linear gradient in methanol and methyl tert-butyl ether); ^b^: the spectral fine structure; ^c^: the ratio of the height of the longest wavelength absorption peak (III) and that of the middle absorption peak (II); ^d^: the ratio of the *cis* peak (AB) and the middle absorption peak (II); ^e^: not applicable; ^f^: not calculated; and ^g^: not detected.

**Table 3:** The carotenoid and chlorophyll content of the *S. obliquus* oleoresin, commercial diet, and cecal content of rats after the intake of oleoresin with different concentrations of microalgae pigments. Different superscript letters on the same line indicate statistical differences (*p* < 0.05) between the experimental groups.

Peak	Pigments	The Pigment Content of the Fecal Sample (µg·g^−1^)							
		*S. oliquus* Oleoresin	Commercial Diet	0 (Control)	75 *	150 *	300 *	450 *	600 *
1	all-*trans*-violaxanthin	61.52 ± 0.52	nd	nd	nd	nd	nd	nd	nd
2	9-*cis*-violaxanthin	49.53 ± 0.39	nd	nd	nd	nd	nd	nd	nd
3	15-*cis*-lutein	81.30 ± 1.19	nd	nd	nd	nd	0.20 ± 0.24	0.21 ± 0.31	0.22 ± 0.99
4	hydroxychlorophyll *a*	136.43 ± 0.41	nd	nd	nd	nd	nd	nd	nd
5	chlorophyll *b*	84.87 ± 1.26	nd	nd	nd	nd	nd	nd	nd
6	13-*cis*-lutein	65.25 ± 1.48	nd	nd	nd	nd	nd	nd	0.21 ± 0.30
7	all-*trans*-lutein	622.98 ± 1.20	1.60 ± 0.25	0.46 ± 0.36	0.48 ± 0.60	0.45 ± 0.49	1.18 ± 0.30	0.93 ± 0.33	2.53 ± 0.14
8	all-*trans*-zeaxanthin	290.13 ± 0.33	1.46 ± 1.10	1.52 ± 1.03	1.65 ± 1.60	2.03 ± 0.23	1.80 ± 1.78	2.48 ± 1.09	3.15 ± 1.10
9	chlorophyll *a*	4459.81 ± 2.17	nd						
10	9-*cis*-lutein	52.53 ± 0.30	nd	nd	nd	nd	0.13 ± 0.08	0.17 ± 0.20	0.23 ± 0.89
11	canthaxanthin	49.46 ± 0.40	nd	nd	nd	nd	nd	nd	nd
12	chlorophyll *a*′	136.14 ± 6.8	nd	nd	nd	nd	nd	nd	nd
13	9-*cis*-zeaxanthin	70.74 ± 1.37	0.80 ± 0.29	0.28 ± 0.83	0.30 ± 0.58	0.24 ± 47	0.33 ± 0.99	0.34 ± 0.70	0.87 ± 0.81
14	2′-dehydrodeoxymyxol	125.32 ± 0.24	nd	nd	nd	nd	nd	nd	nd
15	5,6-epoxy-β-carotene	56.60 ± 0.33	nd	nd	nd	nd	nd	nd	nd
16	all-*trans*-β-cryptoxanthin	55.49 ± 0.17	nd	nd	nd	nd	0.27 ± 0.51	0.28 ± 0.69	0.42 ± 0.50
17	all-*trans*-echinenone	309.93 ± 0.31	nd	nd	nd	nd	nd	nd	nd
18	hydroxypheophytin *a*	nd	0.18 ± 0.51	0.33 ± 0.39	0.24 ± 1.87	0.25 ± 1.03	0.31 ± 0.60	0.64 ± 0.84	0.84 ± 0.77
19	9-*cis*-echinenone	121.31 ± 0.23	nd	nd	nd	nd	nd	nd	nd
20	all-*trans*-β-carotene	264.66 ± 0.27	nd	nd	nd	nd	nd	nd	nd
21	pheophytin *a*	162.21 ± 6.53	0.14 ± 0.82	1.73 ± 0.13	1.75 ± 0.86	1.86 ± 0.38	2.03 ± 1.98	2.51 ± 0.39	3.17 ± 0.19
22	9-*cis*-β-caroten	73.09 ± 0.26	nd	nd	nd	nd	0.19 ± 0.92	0.21 ± 0.31	0.32 ± 0.60
	Total carotenoids	2349.77 ± 1.11	3.85 ± 0.57	1.98 ± 0.62 ^a^	2.43 ± 1.04 ^b^	2.72 ± 0.07 ^c^	4.09 ± 1.01 ^d^	4.62 ± 0.30 ^e^	7.94 ± 0.60 ^f^
	Total chlorophylls	4979.45 ± 2.28	0.32 ± 0.55	2.06 ± 0.17 ^a^	1.98 ± 0.50 ^a^	2.11 ± 0.59 ^b^	2.34 ± 0.49 ^c^	3.15 ± 0.22 ^d^	4.01 ± 0.31 ^e^
	Total pigments	7329.09 ± 1.56	4.17 ± 0.68	4.04± 0.82 ^a^	4.44 ± 0.54 ^b^	4.83 ± 0.19 ^c^	6.43 ± 0.60 ^d^	7.77 ± 0.70 ^e^	11.95 ± 0.34 ^f^

*: Concentration of microalgae pigments present in oleoresin ingested daily (µg·kg^−1^bw·d^−1^). nd: not detected.

**Table 4:** Effects of non-microalgal origin pigments and biomass on gut microbiota.

Pigments/Biomass	Effects	Reference
Lutein; zeaxanthin	Pronounced effect on gut microbiota modulation	[42]
β-Carotene	↑ Bacteroidetes, ↑ Proteobacteria, ↓ harmful bacteria, ↑ Bifidobacterium, Collinsella (intermediate), and Lactobacillus (high doses)	[43]
Lycopene	↓ Proteobacteria, ↑ Bifidobacterium, and Lactobacillus	[44]
Carotenoids (fruits and vegetables)	↑ SCFAs in gut and feces; ↑ butyrate production; modulate IgA production; and prevent/delay dysbiosis	[6,45,46]
β-Cryptoxanthin	No significant changes after 8 weeks of supplementation (3 mg/day)	[47]
Carotenoids (general)	↑ Butyrate production	[6]
Carotenoids (general)	Regulation of immunoglobulin A (IgA) production and the prevention or delay of dysbiosis	[48]
Chlorophylls	↓ Firmicutes and ↑ Bacteroidetes	[4]
Chlorophyll-rich extract	↑ Akkermansia (linked to glucose metabolism)	[49]
Spirulina biomass	↑ SCFAs (acetate, propionate, butyrate, hexanoate, isovalerate, and isobutyrate); ↑ Lachnospiraceae, Lactobacillus, and Bifidobacterium (due to fiber and phenolics)	[50]
Pigments (general)	↑ Antioxidant activity in intestinal lumen; beneficial microbiota modulation	[51,52]
Pigments (general)	May modulate GI transit time, pH, antimicrobial peptides, and secretory IgA	[36]

## Data Availability

The original contributions presented in the study are included in the article, further inquiries can be directed to the corresponding author.
